# Characterization of the complete chloroplast genome of *Buddleja alternifolia* (Buddleiaceae)

**DOI:** 10.1080/23802359.2019.1679050

**Published:** 2019-10-21

**Authors:** Guoqi Zheng, Jie Ma, Juan Yang, Xing Xu

**Affiliations:** a School of life science, Ningxia University, Yinchuan, China;; b Breeding Base for State Key Laboratory of Land Degradation and Ecological Restoration in Northwest China, Ningxia University, Yinchuan, China

**Keywords:** *Buddleja alternifolia*, chloroplast genome, phylogenetic tree

## Abstract

The complete chloroplast genome sequence of *Buddleja alternifolia,* a perennial garden plant and common medicinal plant is widely distributed in west China. The plastome is 154,357 bp in length, with one large single copy region of 85,406 bp, one small single copy region of 18,071 bp, and two inverted repeat (IR) regions of 25,440 bp. It contains 130 genes, including 85 protein-coding genes, 8 ribosomal RNA, and 37 transfer RNA. Phylogenetic tree shows that *B. alternifolia* formed one clade with *Buddleja colvilei* and *Buddleja sessilifolia*. The published plastome of *B. alternifolia* provides significant insight for elucidating the phylogenetic relationship of taxa genus *Buddleja*.

*Buddleja* alternifolia Maxim. belongs to genus *Buddleja* of the Buddleiaceae. Members of this genus are widely distributed in west China, are well known as perennial garden plants (Guan and Chen 2006) and medicinal plants (Cai et al. 2015). The information about *B. alternifolia* was focussed on tissue culture (Liu et al. 2009), seed germination and the establishment of seedlings (Han et al. 2013) and drought resistance physiology (Yan et al. 2017). In the last decades, the use of *B. alternifolia* in landscaping has led to a rapid increase in the information available on the tissue rapid propagation of *B.* *alternifolia*. However, due to anthropogenic over-exploitation and decreasing distributions, this species needs urgent conservation. Knowledge of the genetic information of this species would contribute to the formulation of protection strategy. In this study, we assembled and characterized the complete chloroplast (cp) genome sequence of *B.* alternifolia based on high-quality pair-end sequencing data.

Fresh leaves of *B. alternifolia* were collected from Helanshan mountain (Yichuan, Ningxia, China; coordinate: 105°59′E, 38°40′N). Dried and kept in silica gel for DNA extraction, and then stored in the Herbarium of the College of Life Science, Ningxia University with the accession number of ND190619001. Total genomic DNA was extracted with a modified CTAB method (Doyle and Doyle 1987). First, we obtained 10 million high quality pair-end reads for *B. alternifolia*, and after removing the adapters, the circular genome was assembled in the toolkit GetOrganelle v1.6.2 (Camacho et al. 2009; Bankevich et al. 2012; Langmead and Salzberg 2012; Wick et al. 2015; Jin et al. 2018). The complete chloroplasts genome sequence of *B. colvilei* was used as a reference. Plann v1.1 (Huang and Cronk 2015) and Geneious v11.0.3 (Kearse et al. 2012) were used to annotate the chloroplasts genome and correct the annotation. The complete cp genome sequence was deposited in GenBank under accession number MN395662.

The *B. alternifolia* cp genome is 154,357 bp in length, exhibits a typical quadripartite structural organization, consisting of a large single-copy (LSC) region of 85,406 bp, two inverted repeat (IR) regions of 25,440 bp and a small single-copy (SSC) region of 18,071 bp. The cp genome contains 130 complete genes, including 85 protein-coding genes (85 PCGs), 8 ribosomal RNA genes (8 rRNAs), and 37 tRNA genes (37 tRNAs). Most genes occur in a single copy, while 17 genes occur in double, including 4 rRNAs (4.5S, 5S, 16S, and 23S rRNA), 7 tRNAs (trnA-UGC, trnI-CAU, trnI-GAU, trnL-CAA, trnN-GUU, trnR-ACG, and trnV-GAC), and 6 PCGs (*rps7*, *ndhB*, *ycf2*, *ycf15*, *rpl2* and *rpl23*). The overall GC content of cp DNA is 40.0%, while the corresponding values of the LSC, SSC, and IR regions are 36.1%, 32.2%, and 42.2%.

In order to further clarify the phylogenetic position of *B. alternifolia*, plastome of six representative species were obtained from NCBI to construct the plastome phylogeny, with *Hemsleya lijiangensis* as an outgroup. All the sequences were aligned using MAFFT v.7.313 (Katoh and Standley 2013) and maximum likelihood phylogenetic analyses were conducted using RAxML v.8.2.11 (Stamatakis 2014). The phylogenetic tree shows that *Buddleja* colvilei clustered together with *Buddleja* sessilifolia. and then formed one clade with *B.* *alternifolia* (Figure 1).

**Figure 1.**
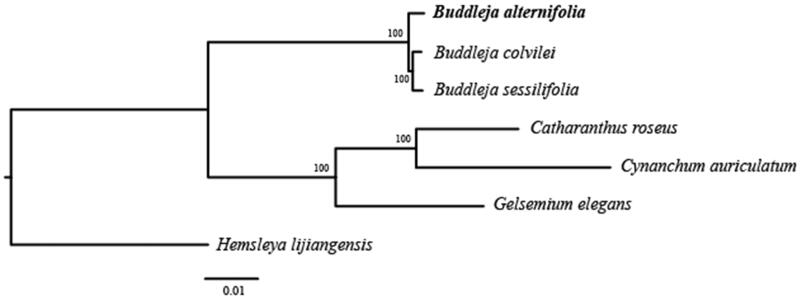
Phylogenetic relationships of *B. alternifolia* using whole chloroplast genome. GenBank accession numbers: *Buddleja colvilei* (NC_042766); *Buddleja sessilifolia* (NC_042767); *Catharanthus roseus* (KC561139); *Cynanchum auriculatum* (KU900231); *Gelsemium elegans* (MH327990); *Hemsleya lijiangensis* (NC_039653).

In summary, the complete cp genome from this study provides significant insight for elucidating the phylogenetic relationship of taxa genus *Buddleja*.

## References

[CIT0001] Bankevich A, Nurk S, Antipov D, Gurevich AA, Dvorkin M, Kulikov AS, Lesin VM, Nikolenko SI, Pham S, Prjibelski AD, Pyshkin AV, et al. 2012 SPAdes: a new genome assembly algorithm and its applications to single-cell sequencing. J Comput Biol. 19:455–477. 22506599 10.1089/cmb.2012.0021 PMC3342519

[CIT0002] Cai L, Li B, Xiao YH,, Dong JX 2015 Chemical constituents from stems and leaves of *Buddleja lindleyana* Fort. J Int Pharmacy Res. 42:634–636.

[CIT0003] Camacho C, Coulouris G, Avagyan V, Ma N, Papadopoulos J, Bealer K, Madden TL 2009 BLAST+: architecture and applications. BMC Bioinformatics. 10:421. 20003500 10.1186/1471-2105-10-421 PMC2803857

[CIT0004] Doyle JJ, Doyle JL 1987 A rapid DNA isolation procedure for small quantities of fresh leaf tissue. Phytochem Bull. 19:11–15.

[CIT0005] Guan WL, Chen X 2006 Preliminary studies on ornamental germplasm resources of genus *Buddleja* L. and its utiliaztion. Southwest China J Agri Sci. 19:371–376.

[CIT0006] Han YY, Ye YH, Luosang WM, Baima DJ, Pu Z 2013 Effect of different substrates on cutting propagation of *Buddleja alternifolia*. Hubei Agri Sci. 52:1868–1871.

[CIT0007] Huang DI, Cronk Q 2015 Plann: a command-line application for annotating plastome sequences. Appl Plant Sci. 3:1500026. 10.3732/apps.1500026 PMC4542940 26312193

[CIT0008] Jin JJ, Yu WB, Yang JB, Song Y, Yi TS, Li DZ 2018 Get Organelle: a fast and versatile toolkit for accurate de novo assembly of organelle genomes. bioRxiv. 256479. doi:10.1101/256479 PMC7488116 32912315

[CIT0009] Katoh K, Standley DM 2013 MAFFT multiple sequence alignment software version 7: improvements in performance and usability. Mol Biol Evol. 30:772–780. 23329690 10.1093/molbev/mst010 PMC3603318

[CIT0010] Kearse M, Moir R, Wilson A, Stones-Havas S, Cheung M, Sturrock S, Buxton S, Cooper A, Markowitz S, Duran C, et al. 2012 Geneious basic: an integrated and extendable desktop software platform for the organization and analysis of sequence data. Bioinformatics. 28:1647–1649. 22543367 10.1093/bioinformatics/bts199 PMC3371832

[CIT0011] Langmead B, Salzberg SL 2012 Fast gapped-read alignment with Bowtie 2. Nat Methods. 9:357–359. 22388286 10.1038/nmeth.1923 PMC3322381

[CIT0012] Liu JF, Wu JH, Li YH 2009 Tissue culure and rapid propagation of Buddleja alternifolia Maxim. Plant Physiol Commun. 45:161.

[CIT0013] Stamatakis A 2014 RAxML version 8: a tool for phylogenetic analysis and post-analysis of large phylogenies. Bioinformatics. 30:1312–1313. 24451623 10.1093/bioinformatics/btu033 PMC3998144

[CIT0014] Wick RR, Schultz MB, Zobel J, Holt KE 2015 Bandage: interactive visualization of de novo genome assemblies. Bioinformatics. 31:3350–3352. 26099265 10.1093/bioinformatics/btv383 PMC4595904

[CIT0015] Yan JW, Li CX, Cui Z, Liu Y 2017 Effects of cadmium on growth, cadmium accumulation and photosynthetic physiology of *Buddleja alternifolia* Maxim. seedlings under drought stress. Acta Ecol Sin. 37:7242–7250.

